# Effect of vitamin E (Tri E^®^) on antioxidant enzymes and DNA damage in rats following eight weeks exercise

**DOI:** 10.1186/1475-2891-10-37

**Published:** 2011-04-23

**Authors:** Noor Aini Abd Hamid, Mohd A Hasrul, Rusdiah J Ruzanna, Ibrahim A Ibrahim, Prasamit S Baruah, Musalmah Mazlan, Yasmin Anum Mohd Yusof, Wan Zurinah Wan Ngah

**Affiliations:** 1 Division of Basic Medical Sciences, Cyberjaya College of Medical Sciences, No 3410, Jalan Teknokrat 3, 63000 Cyberjaya, Selangor Darul Ehsan, Malaysia; 2 Department of Biochemistry, Universiti Kebangsaan Malaysia, Jalan Raja Muda Abdul Aziz 50300 Kuala Lumpur, Malaysia

## Abstract

**Background:**

Exercise is beneficial to health, but during exercise the body generates reactive oxygen species (ROS) which are known to result in oxidative stress. The present study analysed the effects of vitamin E (Tri E^®^) on antioxidant enzymes; superoxide dismutase (SOD), glutathione peroxidase (GPx), catalase (Cat) activity and DNA damage in rats undergoing eight weeks exercise.

**Methods:**

Twenty four *Sprague-Dawley *rats (weighing 320-370 gm) were divided into four groups; a control group of sedentary rats which were given a normal diet, second group of sedentary rats with oral supplementation of 30 mg/kg/d of Tri E^®^, third group comprised of exercised rats on a normal diet, and the fourth group of exercised rats with oral supplementation of 30 mg/kg/d of Tri E^®^. The exercising rats were trained on a treadmill for 30 minutes per day for 8 weeks. Blood samples were taken before and after 8 weeks of the study to determine SOD, GPx, Cat activities and DNA damage.

**Results:**

SOD activity decreased significantly in all the groups compared to baseline, however both exercised groups showed significant reduction in SOD activity as compared to the sedentary groups. Sedentary control groups showed significantly higher GPx and Cat activity compared to baseline and exercised groups. The supplemented groups, both exercised and non exercised groups, showed significant decrease in Cat activity as compared to their control groups with normal diet. DNA damage was significantly higher in exercising rats as compared to sedentary control. However in exercising groups, the DNA damage in supplemented group is significantly lower as compared to the non-supplemented group.

**Conclusions:**

In conclusion, antioxidant enzymes activity were generally reduced in rats supplemented with Tri E^® ^probably due to its synergistic anti-oxidative defence, as evidenced by the decrease in DNA damage in Tri E^® ^supplemented exercise group.

## Background

Exercise is beneficial for the maintenance of a good health but it generates reactive oxygen species (ROS) that may result in oxidative stress [1]. ROS are continuously produced in the normal process of cellular metabolism, but in healthy individuals these are destroyed immediately well within the body's antioxidant defense system. Physical activity increases the generation of ROS in several ways. Two to five percent of oxygen used in the mitochondrial oxidative phosphorylation forms ROS. As the oxidative phosphorylation increases in response to exercise due to increased oxygen consumption, there will be a concomitant increase in free radicals.

Potential sources of ROS during exercise include leakage of electrons from the mitochondrial electron transport chain [2], enhanced purine oxidation, damage to iron-containing proteins, and disruption of Ca^2+ ^homeostasis [3]. Other sources of free radicals that increase with exercise include prostanoid metabolism, xanthine oxidase, NAD(P)H oxidase, and several secondary sources, such as the release of radicals by macrophages recruited to repair damaged tissue [4]. Hence, exercise can produce an imbalance between ROS and antioxidants, which is referred to as oxidative stress.

Oxidative stress defined as the imbalance in the oxidants and antioxidants, in favour of the oxidants potentially leading to cellular damage [5]. Oxidative damage results when the generation of ROS produced exceeds the cellular capacity to destroy them to protect or repair it. ROS lead to alterations in membrane protein structure and also brings changes in enzymatic activity [6]. These events may promote damage to cells by causing alterations in mitochondrial and sarcoplasmic reticular membranes and breakdown of lysosomal membranes. An increase in ROS production may occur during and after exercising by an increase of oxygen consumption, increase of catecholamine levels, lactic acid production, elevated rate of hemoglobin auto oxidation and hyperthermia [7-10]. If the free radical generation is greater than the cell's ability to neutralise them, the radicals will attack cellular components, especially membranous lipids. They initiates a chain reaction involving oxidation of membranous lipids called lipid peroxidation, which leads to generation of more toxic radicals which may harm other cellular components [11].

Antioxidant defence system comprises of enzymes such as catalase, superoxide dismutase, glutathione peroxidase and non-enzymatic antioxidants including vitamin A, vitamin C, vitamin E, ubiquinone and flavonoids. Antioxidants are molecules which interact with ROS and scavenge the free radicals before cellular vital molecules are damaged preventing cellular damage and disease. Vitamin E, a potent naturally occurring lipid-soluble antioxidant possesses the ability to directly quench free radicals and function as a membrane stabilizer. It protects critical cellular structures against the damage from oxygen free radicals and reactive products of lipid peroxidation. The protective effect of vitamin E supplementation against exercise-induced oxidative stress has been reported in humans and rats [12-14].

Vitamin E (Tri E^®^) used in this study was tocotrienol-rich fraction (TRF) palm oil which contains 70% δ-tocotrienol and 30% α-tocopherol. The TRF from palm oil proved to be a more economical and efficient substitute for alpha-tocopherol, significantly inhibited oxidative damage in-vitro to both lipids and proteins in rat brain mitochondria. Studies have shown the ability of vitamin E supplementation to reduce oxidative stress or muscle damage caused by exercise [11]. TRF deficiency can increase free-radical induced tissue injury to levels comparable to those found after exercise, so an adequate status of TRF is important for maintaining membrane integrity during exercise [15,16].

In this research, training protocols with rats running on the specially designed treadmill 30 minutes per day for eight weeks were designed to simulate exercise conditions common to rat. The aim of the study was to demonstrate the oxidative stress and DNA damage due to the treadmill exercise protocols on rats and the effect of supplementation with Tri- E^®^.

## Methods

### Animals and exercise training protocol

Twenty four male rats (weighing 320-370 gm) were obtained from the Animal House, Universiti Kebangsaan Malaysia. They were maintained at 23°C. The Animal Ethical Committee of UKM (UKMAEC) approved the experimental procedure of this study. The rats were divided into four groups: sedentary without supplements, sedentary with Tri E^® ^supplements, exercise without supplements and exercise with Tri E^® ^supplements. Tri E^® ^was purchased from Golden Hope Company and consists of 70% tocotrienols (α,δ and γ) and 30% α-tocopherol. Supplementation protocol is 30 mg/kg/d of Tri E^® ^by oral gastric tube [17]. A total of twelve rats were trained to run on a treadmill (Inbramed, KT-300); at 0.3 km/h up to 0.5 km/h for 30 minutes with each rat individually on the treadmill. The exercise training protocol was performed 7 days per week, 30 minutes per day, over 8 weeks between 8.00am to 10am. Rats were not fasting during the exercise. Blood was obtained from the orbital sinus and collected in heparinised test tubes to prevent from coagulation and the plasma was then separated. Red blood cells (RBC) antioxidant enzymes activity were measured, while whole blood was used in the Comet assay to determine DNA damage before and after 8 weeks of training.

### SOD activity

RBC SOD activity was determined according to the method of Beyer and Fridovich [18]. The amount of hemoglobin that inhibits the rate of nitro blue tetrazolium reduction by 50% is defined as one unit of SOD.

### GPx activity

RBC GPx activity was measured spectrophotometrically by the method of Paglia and Valentine [19]. H_2_O_2 _was added to a medium phosphate buffer, EDTA, NADPH, GSH and erythrocyte hemolysate where the change in absorbance of the system was read at 340 nm.

### CAT activity

RBC CAT activity was described by Aebi in 1984 [20]. The decomposition of H_2_O_2 _by CAT enzyme was read directly by the decrease in absorbance at 240 nm. The difference in absorbance per unit time was measured as CAT activity.

### Comet assay

**Figure 1:**
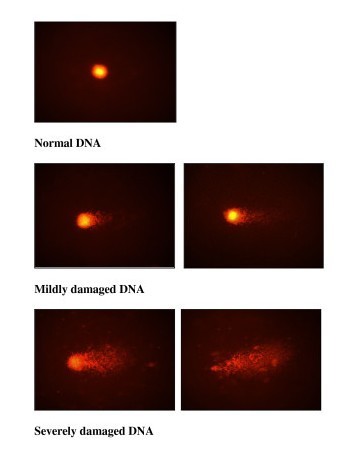
**Figure 1 shows normal DNA and the different degrees of DNA damage as observed in comet assay**.

The Comet assay is a sensitive technique to detect DNA damage in a single cell. Comet assay was determined by using a fluorescence microscope following method by Singh et al. [21]. Whole blood was used for the comet assay. Staining with 50 μL of ethidium bromide. The tests were performed in duplicates, per individual for a total of 500 cells, and mean comet length was calculated. Images of selected nuclei from each sample were analysed. The comet measurements used for analysis were tail length, percentage DNA in tail, tail moment, and proportion of cells. DNA damage was calculated based on percentage of cells over total number of cells scored. The degree of DNA damage determined is as shown in (Figure 1).

## Statistical Analysis

The parameter values were all expressed as the mean ± standard deviation. Significant differences among the groups were determined by one-way ANOVA using SPSS 12.0 software package programme. The results were considered significant if the value is p < 0.05.

## Results

### Effect of exercise and Tri E^® ^supplementation on SOD activity

**Figure 2:**
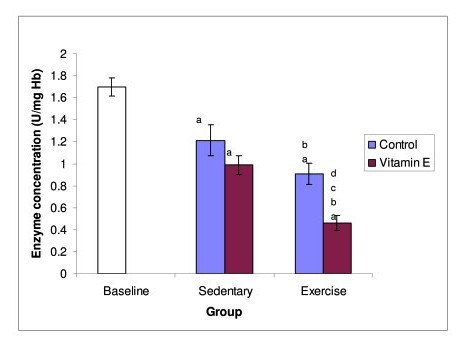
**Figure 2 shows the effect of exercise and Tri E^® ^supplementation on SOD activity**. SOD activity decreases significantly in the sedentary supplemented group and non supplemented exercise groups. Exercise supplemented group shows further decrease in its activity. a = significantly (P < 0.05) lower when compared to baseline b = significantly (P < 0.05) lower when compared to sedentary, control group c = significantly (P < 0.05) lower when compared to sedentary, vitamin E group d = significantly (P < 0.05) lower when compared to exercise, control group

The results showed that Superoxide dismutase (SOD) activity (Figure 2) decreased significantly in all four experimental groups compared to baseline. However, both exercise groups showed significant reduction in SOD activity as compared to the sedentary groups.

### Effect of exercise and Tri E^® ^supplementation on GPx activity

**Figure 3:**
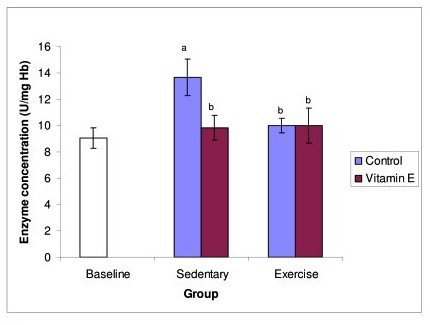
**Figure 3 shows the effect of exercise and Tri E^® ^supplementation on GPx activity**. GPx activity increased significantly after 8 weeks of exercise and decreases significantly with Tri E^® ^supplementation. However, no change in GPx activity in both the exercising groups. a = significantly (P < 0.05) higher when compared to baseline b = significantly (P < 0.05) lower when compared to sedentary, control group

Glutathione peroxidase (GPx) activity (Figure 3) increased significantly in sedentary control group compared to baseline. However, in the supplemented sedentary group and both the exercise groups its activity decreased significantly compared to sedentary control group, but no change noted between the exercise groups.

### Effect of exercise and Tri E^® ^supplementation on CAT activity

**Figure 4:**
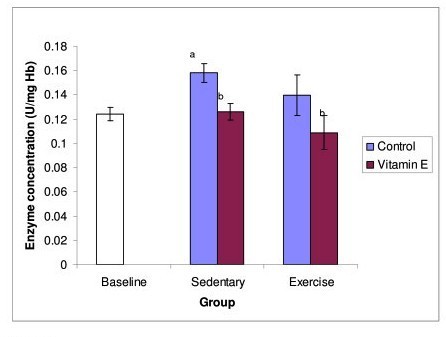
**Figure 4 shows the effect of exercise and Tri E^® ^supplementation on Cat activity**. Cat activity was significantly decreased in the supplemented groups, both exercising and non exercising groups. a = significantly (P < 0.05) higher when compared to baseline b = significantly (P < 0.05) lower when compared to sedentary, control group

Catalase (CAT) activity (Figure 4) increased significantly in sedentary control group compared to baseline. The sedentary and exercise groups supplemented with vitamin E showed significant decrease in catalase activity as compared to sedentary control group.

### Effect of exercise and Tri E^® ^supplementation on DNA damage

**Figure 5:**
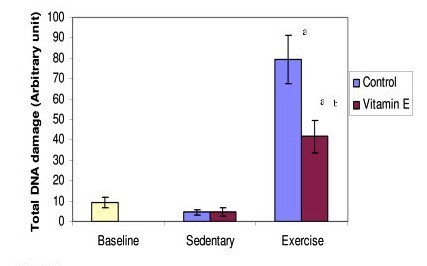
**Figure 5 shows the effect of exercise and Tri E^® ^supplementation on DNA damage**. There was a significant increase in the DNA damage in exercising groups both supplemented and non supplemented, as compared to the sededentary groups, but the supplemented exercising group showed significant reduction in DNA damage as compared to the non supplemented exercise group. a = significantly (P < 0.05) higher when compared to sedentary group b = significantly (P < 0.05) lower when compared to control in exercise group

DNA damage (Figure 5) was significantly higher in exercising rats as compared to sedentary control; however, the DNA damage in supplemented exercise group is significantly lower as compared to the non-supplemented exercise group.

## Discussion

Dietary antioxidants play a crucial role in preventing the toxic effects of endogenous reactive oxygen species and studies support a protective role of vitamin E against oxidative stress induced by exercise [22]. In rats, deficiency in vitamin E can increase the susceptibility of these animals to oxidative stress induced toxicity and human studies have shown that vitamin E supplementation reduces the oxidative stress and lipid peroxidation induced by exercise [23].

Most studies suggest that stressors like exercise increases the free radical generation in the body which in turn stimulates the increased production of antioxidant enzymes GPx and SOD [24]. Antioxidant enzymes SOD, CAT and GPx prevent the cellular toxic effects of free radicals, generated during oxidative stress by scavenging ROS immediately at the site of their production. A balance between antioxidants and oxidant production ensures a protective cellular environment.

SOD functions as one of the primary enzymatic antioxidant defense against highly reactive superoxide radicals. It catalyses the dismutation of superoxide into oxygen and H_2_O_2_. SOD converts superoxide to H_2_O_2_, which is in turn catabolised to water mainly by CAT, preventing the formation of the highly reactive hydroxyl radical (OH^**.- **^) [25].

Previous studies reported that increased levels of SOD activity in blood and muscle at rest are common in trained individuals, and SOD activity is increased in response to exercise interventions in a trained population [11].

Endurance training has been shown to increase SOD activity in skeletal muscle [26,27]. However, not all studies are consistent with these conclusions. This is consistent with our results which showed that the level of SOD is decreased in exercise group as compared to sedentary group. The reduction in SOD activity is unclear, but it could be due to the age related changes and also probably due to the activity of rats have been reduced during the 8 wks of the confinement in the cage. In reduce physical activity of rats the oxygen radical produced will be less and thus reducing the SOD activity. Supplementation with Tri E^® ^showed a further decrease in SOD activity as compared to non-supplemented group. Tri E^® ^may have induced adaptive changes in the antioxidative defenses in order to compensate for greater free radical generation during exercise [22,28]. The ROS may have been removed by the antioxidant action which then reduced the need for the SOD to be induced.

In GPx catalysed reaction, glutathione is oxidised inactivating H_2_O_2 _to H_2_O. GPx belongs to peroxidase class of enzymes found in the erythrocytes of mammals that prevents lipid peroxidation of the cellular membrane. GPx reduces lipid hydroperoxides generated during the lipid peroxidation to their corresponding alcohols and reduces free hydrogen peroxide to water [29].

It has been reported that there is a significant correlation of GPx activity and weekly training in runners [30]. Ortenblad et al. observed no difference in erythrocyte GPx or glutathione reductase activity between trained and untrained subjects, however muscle activities of these enzymes were higher in the trained subjects [31]. In correspondence to our study, we found a significant decrease in the level of GPx activity in exercise group compared to sedentary group, with or without supplementation. GPx activity was also reported to decrease as a result of adaptive changes in trained rats compared to untrained rats [32]. Vitamin E has glutathione(GSH) sparing effect. By supplementation with Tri E^® ^GSH is spared. GPx uses GSH and when there is sparing of GSH, action of GPx is not affected by exercise, so we would expect no change in the exercising group with and without vitamin e supplementation.

Reactive oxygen species itself can act as a signal during exercise which upregulates the expression of GPx to prevent the oxidative stress. Catalase is widely distributed in the cell, with the majority of the activity occurring in the mitochondria and peroxisomes. Catalase activity in response to a single bout of exercise is variable. Catalase activity undergoes adaptive changes during the exercise [33,34].

Reports have also depicted that there is no difference in catalase activity levels following marathon running [12]. Our results also showed that the level of catalase activity decreased between the exercise group compared to sedentary group. The results also showed that Cat activity decreased in supplemented Tri E^® ^compared to non-supplemented and the reason being Tri E^® ^may have taken over oxidative defense and thus reduced the induction of Cat [21]. The mechanism is unclear, however it has been found that tocotrienol in Tri E^® ^has effect on gene expression of antioxidant response elements.

Comet assay showed that exercise groups had significantly increased DNA damage as compared to the sedentary group. Oxidative tissue damage in vitamin E deficient animals is exacerbated by endurance training and, conversely, it is reduced by high-dose vitamin E supplementation; also, preliminary studies in humans have demonstrated antioxidant protection by high-dose vitamin E supplementation [35].

This is consistent with the study done by Tsai et al, 2001, on human study who reported an increase in DNA damage 24 hour post exercise that persisted through day 7 in response to a 42 km run (average run time 3 hours) [36]. Supplementation with Tri E^® ^had decreased the DNA damage significantly. This is in accordance with the human studies done earlier which found that supplementation with vitamin E for 8 weeks was effective in reducing DNA damage after an incremental exercise test to exhaustion in healthy non smokers aged between 29-34 years [37]. Supplementation with vitamin E before the exercise seemed to have the good effect, leading the investigators to conclude that vitamin E prevents exercise-induced DNA damage [13]. Vitamin E prevents the leakage of the cellular enzymes and content due to ROS [35].

## Conclusions

In conclusions, this study showed that eight week exercise training had adaptive effects on antioxidant enzymes activity and supplementation with Tri E^® ^showed a synergistic effect by further reducing their activity as proven by the reduction in DNA damage in supplemented exercising rats as compared to the non-supplemented group.

## Competing interests

The authors declare that they have no competing interests.

## Authors' contributions

MHA and RRJ did the experimental work, while IAI and PSB drafted the manuscript. MMZ and YAMY contributed to the study design. NAH and WZWN took part in planning the study design, supervising the study design, contributed to the analysis and writing of manuscript, edited the paper, revising it critically for important intellectual content and provided the final version. All authors read and approved the final manuscript.
